# Profiles of Recruits Entering Army Basic Training in New Zealand

**DOI:** 10.1093/milmed/usac090

**Published:** 2022-04-12

**Authors:** Narelle Hall, Maria Constantinou, Mark Brown, Belinda Beck, Michael Steele, Jacques Rousseau, Suzanne Kuys

**Affiliations:** School of Allied Health, Faculty of Health Sciences, Australian Catholic University, Banyo, QLD 4014, Australia; School of Allied Health, Faculty of Health Sciences, Australian Catholic University, Banyo, QLD 4014, Australia; Faculty of Health Sciences, Australian Catholic University, Banyo, QLD 4014, Australia; School of Allied Health Sciences, Exercise and Sport, Gold Coast Campus, Griffith University, Southport, QLD 4215, Australia; School of Allied Health, Faculty of Health Sciences, Australian Catholic University, Banyo, QLD 4014, Australia; Human Performance Cell, Joint Support Group, New Zealand Army, Upper Hutt 5018, New Zealand; School of Allied Health, Faculty of Health Sciences, Australian Catholic University, Banyo, QLD 4014, Australia

## Abstract

**Introduction:**

A high incidence of musculoskeletal injuries is sustained by army recruits during basic training. Describing recruits’ personal, lifestyle, and physical performance characteristics at the entry to training can help identify existing intrinsic risk factors that may predispose some recruits to injury. Identifying modifiable and preventable intrinsic risk factors may contribute to lower recruit injury and associated burdens during the course of basic training. The aim of this study was to therefore describe the profile of New Zealand Army recruits upon entry to basic training using personal, lifestyle, and physical performance characteristics.

**Methods:**

New Zealand Army male and female recruits from two intakes in the same year were invited to participate. Recruits’ data on personal (sex, age, height, and weight), lifestyle (self-reported responses to the Military Pre-training Questionnaire comprising physical and injury history, diet, alcohol, and smoking status) and physical performance characteristics (2.4-km timed run, weight-bearing dorsiflexion lunge test, and the Y Balance Test^TM^ for lower limb dynamic stability) were collected and analyzed.

**Results:**

Participants included 248 New Zealand Army recruits: 228 males (91.9%), 20 females (8.1%), average age of 20.3 ± 2.8 years. Findings indicated 30.9% of recruits reported injury in the 12 months prior to training commencing, with 44.8% of those injuries in the lower limbs. Pre-entry alcohol consumption was higher than recommended and 20.1% of recruits identified as current smokers. Recruits who passed the 2.4-km timed run included 53.8% of males and 28.6% of females. Weight-bearing dorsiflexion lunge test performance was within a normal range (right = 10.3 ± 3.3 cm), however limb asymmetry (>1.5 cm) was present with 30.9% of recruits. For the Y Balance Test^TM^ for dynamic lower limb stability, 70% of female recruits had high posterolateral reach asymmetry (8.1 ± 6.0 cm), while normalized composite reach scores were low (right) for male (92.2 ± 8.1%) and female recruits (89.0 ± 7.5%).

**Conclusions:**

New Zealand Army recruits entering basic training were predominantly active young males, reported few injuries in the previous year, had higher than recommended alcohol consumption and a minority were smokers. The majority of recruits had low aerobic fitness, average ankle dorsiflexion range, and low dynamic lower limb stability. While a number of adverse characteristics identified are potentially modifiable, more research is required to identify an association to musculoskeletal injury risk in New Zealand Army recruits. Describing the profile of recruits entering training, particularly recruits at risk of injury is one of the first steps in injury prevention.

## INTRODUCTION

The incidence of musculoskeletal injuries sustained by military recruits during basic training from western countries is high.^1–4^ Overall musculoskeletal injury incidence may be as high as 86% during infantry basic training^5^ and up to 80% of all injuries occur in the lower limbs.^4,6^ Overuse injury (65%) is more common than acute (35%),^4^ and female military recruits are at two times greater risk of injury than males during army fulltime^7^ and reserve basic training.^8^

Detrimental consequences of recruit lower limb musculoskeletal injuries can include lengthy injury rehabilitation,^9^ subsequent and/or chronic injury,^3,10,11^ injury time loss,^4^ and backsquad (recycled) or discharge from military service.^8,12^ For the military, recruit musculoskeletal injury consequences include work and training time loss, increased demand and cost on health resources,^3,9,12^ rising recruitment and retention costs, and service attrition.^3,12^ In the long term these burdens potentially impact organization effectiveness and operational capability.^3,12^

Specific personal, lifestyle, and physical performance characteristics have been identified as intrinsic (person-related) risk factors which may predispose some recruits to musculoskeletal injury during training. Established recruit personal and lifestyle intrinsic injury risk factors include older age,^13,14^ female sex,^3,8^ pre-existing injury,^4^ and smoking history.^13,14^ Physical performance characteristics such as low pre-entry aerobic fitness^4,6,15^ have also been identified.

Other physical performance characteristics such as low (or high) ankle dorsiflexion range of motion (flexibility) and dynamic lower limb stability (balance) have been associated with musculoskeletal injury risk in sports and trained military populations.^16–18^ However, research with military recruit populations is limited. For example, a study of Australian Army recruits (*n* = 1,093) reported males with restricted ankle dorsiflexion range of motion were 2.5 times more likely to incur lower limb injury, while recruits with higher flexibility (high ankle dorsiflexion range of motion) were up to eight times more likely to incur lower limb injury during training.^19^ Measurement of ankle dorsiflexion range, however, required technical proficiency, additional equipment (T square and fixed meter ruler) and trigonometry calculation.^19^ Investigation of simpler, field- and resource-friendly measurement methods is important for clinicians and researchers establishing baseline dorsiflexion range of motion values in recruit populations.

Poor lower limb dynamic stability (balance) in relation to injury has also been investigated in sports populations. For example, male and female high school basketball players with a Star Excursion Balance Test anterior reach asymmetry greater than 4 cm were 2.5 times more likely to sustain a lower limb injury (*P* < .05). The same study found female basketballers with a normalized composite reach distance less than 94%, were 6.5 times more likely to sustain a lower limb injury (*P* < .05).^20^ Lower limb dynamic stability baseline values and/or risk of injury in military recruits have been assessed in a limited number of studies. Male Brazilian military recruits (*n* = 135) with high Y Balance Test™ (modified Star Excursion Balance Test) posterolateral reach direction asymmetry (≥4.08 cm) were more likely to develop patellofemoral pain over 6 weeks basic training.^21^ Alternatively, no relationship was identified between Y Balance Test™ performance and injury risk in U.S. Army, Airforce, Navy, and Marine recruits.^22^ Establishing clear baseline values of dynamic lower limb stability is warranted in different recruit populations and countries.^18^

Describing recruit personal, lifestyle, and physical performance characteristics at entry to training is important in establishing baseline values and identifying potential intrinsic injury risk factors. Modification of preventable factors may contribute to lower recruit injury and associated burdens. The aim of this cross-sectional study was to describe the profile of male and female New Zealand Army recruits entering basic training across two recruit intakes including personal, lifestyle, and physical performance characteristics.

## METHODS

### Participants

Participants were drawn from the general New Zealand population coming into the New Zealand Army. Prospective recruits volunteer to join the New Zealand Army, pass the New Zealand Defence Force aptitude test (basic reading and writing test), and attest (be sworn in) to the army to commence basic training. Prospective recruits enter the service with varying levels of physical fitness and experience.

Two intakes (approximately 140 per intake) of male and female regular force recruits (≥17 years) were eligible to commence basic training in 2012 at the participating training site. Recruits were provided with study information and an opportunity to ask questions before providing voluntary written informed consent to take part in this study. Included in the study were recruits who attested to the army and provided consent to participate. Recruits who declined consent to participate in the study, declined attestation to the army, or who were returning to training (after week one) due to being backsquadded (recycled) from previous recruit intakes, were excluded.

### Measures

#### Personal characteristics

Sex and age were recorded by Army physical training instructors. Height (cm) and weight (kg) were obtained from The Army Depot personnel list and the local medical database. At entry medicals, height and weight is measured with recruits dressed in training uniform and without footwear.

#### Lifestyle characteristics

Lifestyle characteristics were recorded using the Military Pre-training Questionnaire.^23^ The Military Pre-training Questionnaire is a low-cost, reliable, self-reported, and descriptive questionnaire comprising five domains to assess multiple injury-related risk factors for military basic training recruits.^23^ The five domains are physical activity, injury history, diet, alcohol, and smoking status; each scored separately. The Military Pre-training Questionnaire includes previously validated tools (Leisure-Time Exercise Questionnaire,^24^ the modified Rapid Eating and Activity Assessment of Patients,^25^ Alcohol Use Disorders Identification Test and Consumption questions,^26^ and the Cigarette Dependence Scale-5^27^) with additional items relating to military recruit injury risk.^23^ Sections of the questionnaire have identified British Army infantry recruits at high risk of musculoskeletal injuries undertaking basic training.^4^ The 15-minute questionnaire was issued to recruits by medical administrative staff during week one of basic training with sealed responses placed into a secure box for collection.

Within the Military Pre-Training Questionnaire, the Leisure-Time Exercise Questionnaire reports^24^ pre-entry physical activity level. Expressed in units, the weekly frequency of activity based on metabolic equivalent values for listed exercise categories are summated to provide a total weekly activity score.^24,28^ A total score of ≥24 units indicates active, 14 to 23 units indicates moderately active and <14 units indicates insufficiently active.^28^ Only the responses to the first question of the Leisure-Time Exercise Questionnaire are presented in this study. A modified version of the Rapid Eating and Activity Assessment for Patients^25^ compromised 24 questions to assess recruits’ pre-entry self-reported dietary behaviors.^23^ Questions (scored one to three) were summed out of a total of 72 to provide an estimation of diet quality with higher scores indicating higher diet quality.^29^ The Alcohol Use Disorders Identification Test and Consumption questions (AUDIT-C)^26^ are a modified version of the 10-item AUDIT.^30^ This three-item questionnaire was used to ascertain recruits' pre-entry alcohol consumption.^23^ Questions are summed with scores ranging from 0 to 12; a score of 0 indicates no drinking^26^ while higher scores (>5) suggest a risk of hazardous drinking.^31^ Scores of four or more for males and three or more for females are considered positive.^31^ Smoking status, established from the smoking section of the Military Pre-Training Questionnaire,^23^ was reported as the number and percentage of recruits who identified as current smokers, ex-smokers, and nonsmokers at the entry to training.

#### Physical performance characteristics

Physical performance characteristics were recorded using the 2.4-km timed run, the weight-bearing dorsiflexion lunge test,^32^ and the lower quadrant Y Balance Test™.^33^ All tests were completed in week one of recruit basic training. The 2.4-km timed run was administered by Army physical training instructors and both the weight-bearing dorsiflexion lunge test and the Y Balance Test™ were performed by two trained examiners (physiotherapists and/or one remedial instructor) during the initial medical review periods.

The 2.4-km timed run is a cost-efficient, field-based test of aerobic fitness used with military personnel with slow run times associated with higher musculoskeletal injury risk in male and female recruits.^4,6,15^ The 2.4-km run course is set over tarmac roads inside the military camp and recruits must run the course in the fastest time possible. Recruits are required to pass the 2.4-km timed run at least once during basic training in order to march out (complete training). Results were recorded in minutes and allocated either as a pass or fail depending on age and sex-adjusted grades. A pass grade for New Zealand Army recruits aged 25 years or less for males is 10.5 minutes and females is 12.3 minutes.

**FIGURE 1.**
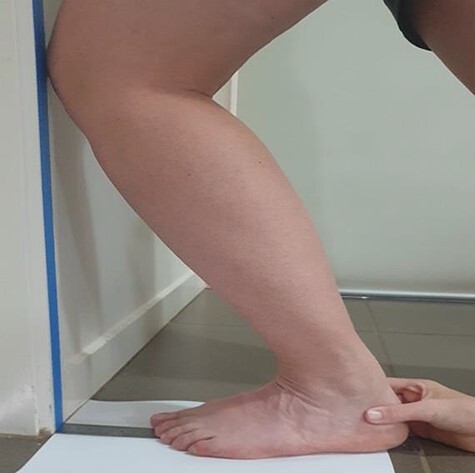
The weight-bearing dorsiflexion lunge test for ankle range of motion.

The weight-bearing dorsiflexion lunge test^32^ measures ankle dorsiflexion range of motion with high or low range (flexibility) associated with greater risk of lower limb musculoskeletal injuries in army recruits.^19^ The test involves standing facing a wall and lunging forward so that the knee touches a vertical line drawn on the wall in front of the recruit (Fig. 1). The foot is progressively moved backward until a maximum lunge is reached while the knee contacts the wall. During the standing lunge, the recruit’s heel was held by the tester to prevent lifting from the floor and the recruit was advised to align their knee with their second toe. The untested back foot was placed on the floor. Up to five tests were allowed and at the maximum lunge point, the tester measured the distance to the wall from the tip of the recruit’s big toe in centimeters (to the nearest 0.1 cm).^32^ Recruits scoring further than 16.1 cm (58º) were classified as high dorsiflexion range of motion and those scoring less than 9.4 cm (34º) were classified as low dorsiflexion range of motion^19^ with a relationship of 1 cm to 3.6º applied.^32,34^ Asymmetry was the difference between right and left lower limb scores (cm). The percentage of recruits with a weight-bearing dorsiflexion lunge asymmetry greater than 1.5 cm (impaired dorsiflexion range) was recorded.^34^ The standing weight-bearing dorsiflexion lunge test for distance is considered time-, cost-, and resource-efficient^32^ and requires low technical proficiency.^35^ The distance method has good intrarater reliability (ICC = 0.98) and low measurement error for novice raters.^35^

The Y Balance Test™ is a modified version of the Star Excursion Balance test for dynamic lower limb stability (balance) which can predict musculoskeletal injury risk in sports^20,36^ and trained military populations.^37^ The Y Balance Test™ is performed standing barefoot on one leg while simultaneously reaching as far as possible with the non-weight-bearing leg over three directions: anterior, posteromedial, and posterolateral.^33^ Up to six practice trials were allowed, followed by three formal trials.^33^ Testing was set up in accordance with recommendations for standardization using the Y Balance Test™ kit equipment (YBT Kit, Move2Perform Evansville, Indiana, US).^33^ The Y Balance Test™ performance was scored as the maximum individual reach right and left for anterior, posteromedial, and posterolateral (to the nearest 0.5 cm) directions. Individual reach asymmetry was calculated as the difference between right and left lower limb scores (cm). Asymmetry scores greater than 4 cm were identified in each direction (>4.0 cm anterior,^20^ ≥ 4.0 cm posteromedial,^36^ and ≥ 4.08 cm posterolateral^21^) because of their ability to predict musculoskeletal injuries in sports and recruit populations.^20,21,36^ Composite reach score (normalized to leg length) was calculated as the summation of the three reach directions (anterior, posteromedial, and posterolateral), divided by three times the lower limb length (measured from anterior superior iliac spine to the distal portion of the medial malleolus (cm) and multiplied by 100 (%).^33^ The number and percent of recruits scoring below the normalized composite reach the cut-off score of 94%^20^ is reported. The Y Balance Test™ has good to excellent intrarater (ICC 0.85-0.91) and interrater reliability (ICC 0.99-1.00).^33^

Descriptive statistics were presented as mean (± standard deviation) and frequencies (%) for combined recruit scores, males and females. Analyses were conducted using IBM SPSS Statistics for Windows (Version 27.0) (IBM Corp. Armonk, New York, USA).

Ethical clearance was granted by the Commander of Training and Doctrine from the New Zealand Defence Force February 2012 (updated 2019) and from Griffith University Human Research Ethics Committee May 2012 (PES/36/11/HREC).

## RESULTS

Participants initially included 281 recruits from two intakes (four platoons per intake). Thirty-three recruits were excluded; including five who did not consent to participate in the study. The final analysis, therefore, included 248 regular force New Zealand Army recruits (228 male, 20 female) with an average age of 20.3 ± 2.8 years. Participants’ characteristics are described in Table I. Responses of the Military Pre-training Questionnaire for physical, injury, diet, alcohol, and smoking history are presented in Table II. The average recruit pre-entry Leisure Time Exercise Questionnaire score was 62.5 ± 27.0 units (62.7 ± 27.4 male, 60.0 ± 23.3 female). Seventy-one (30.9%) recruits reported an injury in the previous 12 months (MPQ Q2-4) with 30 (44.8%) of those in the lower limbs. The average score from the modified Rapid Eating and Activity Assessment for Patients reporting recruit pre-entry diet status was 47.7 ± 6.0 out of a total score of 72 (47.6 ± 6.1 males, 48.9 ± 4.6 females). Recruits average score from the Alcohol Use Disorders Identification Test-Consumption questions was 5.3 ± 3.0 units (5.4 ± 3.0 males, 3.5 ± 2.4 females). Approximately 57% of recruits commencing basic training reported being nonsmokers with 23% reported being ex-smokers and 20% current smokers.

**TABLE I.** Recruit Personal Characteristics

Variable	Total (n = 248)	Males (n = 228, 91.9%)	Female (n = 20, 8.1%)
Age (years)	20.3 (2.8)	20.3 (2.8)	20.7 (3.4)
Under 25	233 (94%)	216 (95%)	17 (85%)
Height (cm)	178.5 (7.3)	179.5 (6.6)	167.6 (5.9)
Weight (kg)	77.9 (11.4)	78.3 (11.3)	73.3 (11.2)
BMI (kg/m^2^)	24.4 (3.1)	24.3 (3.0)	26.0 (3.1)

BMI is Body Mass Index. Data presented as mean (standard deviation) or frequency (%).

**TABLE II.** Recruit Lifestyle Characteristics (Military Pre-training Questionnaire)

Military Pre-training Questionnaire	Total (*n* = 248)	Male (*n* = 228)	Female (*n* = 20)
^a^Physical history (units)	62.5 (27.0)	62.7 (27.4)	60 (23.3)
Injury in the last year	71 (30.9%)	62 (29.4%)	9 (47.4%)
Previous lower limb injury last year	30 (44.8%)	24 (40.7%)	6 (75.0%)
^b^Diet history (score)	47.7 (6.0)	47.6 (6.1)	48.9 (4.6)
^c^Alcohol Status (units)	5.3 (3.0)	5.4 (3.0)	3.5 (2.4)
^d^Smoking Status			
Non-smoker	130 (56.8%)	119 (56.7%)	11 (57.9%)
Ex-smoker	53 (23.1%)	48 (22.9%)	5 (26.3%)
Smoker	46 (20.1%)	43 (20.5%)	3 (15.8%)

Data presented as mean (standard deviation) or frequency (%).

a Leisure-Time Exercise Questionnaire (LTEQ) (*n* = 229),.

b The modified Rapid Eating and Activity Assessment for Patients (REAP) (*n* = 230),.

c Alcohol Use Disorders Identification Test-Consumption (AUDIT-C) (*n* = 230).

d Smoking status (*n* = 229).

Results of physical performance measures for all, male and female recruits for the 2.4-km timed run, weight-bearing dorsiflexion lunge test, and the Y Balance Test™ are presented in Table III. On average, recruit 2.4-km run time was 10.7 ± 1.4 minutes (10.6 min, ± 1.2 male, 13.5 min, ± 1.7 female). There were 53.8% male and 28.6% of female recruits who met the 2.4-km timed run requirements (passed).

**TABLE III.** Recruit Physical Performance Characteristics

Physical measure	Total (*n* = 248)	Male (*n* = 228)	Female (*n* = 20)
2.4-km timed run (seconds)	644.3 (83.6)	633.2 (69.5)	808.5 (104.9)
2.4-km timed run (minutes) (208 M, 14 F)	10.7 (1.4)	10.6 (1.2)	13.5 (1.7)
WBDFLT (cm) (223 M, 20 F)			
Right	10.3 (3.3)	10.4 (3.4)	9.5 (2.7)
Recruits < 9.44 cm (34^°^) (low)	106 (43.6%)	97 (43.5%)	9 (45.0%)
Recruits > 16.1 cm (58^°^) (high)	12 (4.9%)	12 (5.4%)	0 (0.0%)
Left	10.2 (3.2)	10.2 (3.3)	9.4 (2.8)
Recruits < 9.44 cm (34^°^) (low)	108 (44.4%)	99 (44.4%)	9 (45.0%)
Recruits > 16.1 cm (58^°^) (high)	8 (3.3%)	8 (3.6%)	0 (0.0%)
^a^Asymmetry	1.3 (1.4)	1.3 (1.4)	1.4 (1.4)
Asymmetry > 1.5 cm	75 (30.9%)	69 (30.9%)	6 (30.0%)
YBT-LQ absolute reach (cm) (225 M, 20 F)			
Anterior			
Right	61.5 (7.0)	62.0 (6.8)	55.2 (6.7)
Left	62.0 (7.5)	62.5 (7.4)	56.3 (6.6)
^a^Asymmetry	3.1 (2.7)	3.1 (2.6)	3.6 (3.4)
Recruits with asymmetry > 4 cm	63 (25.7%)	56 (24.9%)	7 (35.0%)
Posteromedial			
Right	99.0 (9.4)	99.8 (9.2)	90.4 (6.8)
Left	100.3 (9.5)	101.1 (9.2)	91.8 (8.3)
^a^Asymmetry	4.0 (3.5)	4.0 (3.4)	4.4 (5.3)
Recruits with asymmetry ≥ 4 cm	106 (43.3%)	98 (43.6%)	8 (40.0%)
Posterolateral			
Right	92.1 (10.9)	93.0 (10.7)	82.5 (7.3)
Left	92.6 (11.2)	93.3 (11.1)	85.1 (9.6)
^a^Asymmetry	5.1 (4.0)	4.8 (3.7)	8.1 (6.0)
Recruits with asymmetry ≥ 4.08 cm	126 (51.4%)	112 (49.8%)	14 (70.0%)
YBT-LQ Composite (normalized) (%)			
Right	92.0 (8.1)	92.2 (8.1)	89.0 (7.5)
Recruits < 94% cut-off	149 (60.8%)	133 (59.1%)	16 (80.0%)
Left	92.4 (8.6)	92.6 (8.5)	90.3 (9.7)
Recruits < 94% cut-off	150 (61.2%)	137 (60.9%)	13 (65.0%)

Data presented as mean (standard deviation) or frequency (%).

cm, centimetres, F, Female, km, kilometre, M, Males, *n*, No., Number, WBDFLT, Weight-bearing dorsiflexion lunge test, YBT-LQ, Y Balance Test^TM-^ Lower Quadrant.

a Asymmetry is the absolute difference (cm) between right and left.

## DISCUSSION

This study provided a profile of New Zealand Army recruits entering basic training. Recruits were predominantly males (91.9%), approximately 20 years old (<25 years, 94%) with a normal (healthy) BMI of 24.4 kg/m^2^. A similar proportion of males and females (91.8% and 8.2%, respectively) has been reported in Australian Army full-time recruits (*n* = 12, 077),^8^ and similar (comparable) personal characteristics for age, height, weight, and BMI of male army recruits are reported across militaries in different countries.^8,13^

The majority of New Zealand Army recruits in the current study were active, few reported injuries in the previous year, diet quality was mid-range and most recruits were nonsmokers (57%). Of concern is the number of recruits with preexisting injury, high pre-entry alcohol consumption, and current smokers as these characteristics have shown a higher association to musculoskeletal injury risk during basic training.^4,23^

Preexisting injury has been reported to increase the risk of subsequent or recurrent injury^4^ and possible chronic injury^3,10,11^ for recruits undertaking basic training. In this study, 30.9% of New Zealand Army recruits presented with preexisting injuries. Similar proportions are found with U.S. Army military police recruits (*n* = 2,391, 27.5%),^13^ however a lower percentage of preexisting injuries are reported by British Army infantry recruits (*n* = 1,810, 22.0%).^4^ While preexisting lower limb injury is a well-established recruit intrinsic injury risk factor,^4,13,14^ it remains unclear if recruits with preexisting injury had adequate injury rehabilitation and fully recovered prior to training commencement.^13^ Pre-entry injury outcome status (fully recovered or not) is therefore important to include in future questionnaires to better determine recruit injury risk^4,23^ and identify recruits who could benefit from injury rehabilitation prior to training.

Alcohol and smoking consumption have been associated with physiological and psychosocial injury risk in recruit training populations. Entry-level alcohol consumption in the current study sample was above-recommended cut points of four or more for males and three or more for females,^31^ while one-fifth of recruits (20%) identified as current smokers. Both these lifestyle characteristics have been associated with the development of stress fracture and other health-related factors^38^ including increased social risk-taking behavior.^13^ On the other hand, a recent systematic review of U.S. studies suggests evidence of an association between alcohol consumption and recruit injury is insufficient.^39^ By contrast, smoking is a well-established recruit injury risk factor.^13,14^ Identification of adverse lifestyle factors in recruits is important as they may be modifiable with intervention pre-entry. Additionally, some factors (alcohol, diet, and smoking) could be standardized upon entry as part of the controlled military living and training environment, potentially contributing to a lower risk of recruit injury.

Physical performance characteristics describe New Zealand Army recruits as having slow run times, normal ankle dorsiflexion range of motion (flexibility), and low dynamic lower limb stability. Just over half of male recruits and less than a third of female recruits passed the 2.4-km timed run, providing evidence there is low fitness on entry. Low pre-entry fitness is a significant risk factor for recruit musculoskeletal injury^4,6,15^ and attrition^15^ across multiple basic training populations. For example, in a population of British female recruits, the average 2.4-km run time for noninjured recruits was faster than injured recruits (12 minutes 13 seconds compared with 12 minutes 43 seconds) and for every 10 seconds increase in time, there was an 8.3% increased risk of musculoskeletal injury.^6^ Adherence to 2.4-km timed run-pass requirements or more stringent times is likely to lower musculoskeletal injury rates and associated burdens; the challenge for armies is finding a balance between recruits meeting entry fitness requirements and achieving military entry quotas.

While the majority of New Zealand Army recruits exhibited normal or optimal ankle dorsiflexion range of motion (flexibility), more than 45% of recruits had low (<9.4 cm) or high (>16.1 cm) dorsiflexion range of motion, and approximately 30% of recruits displayed asymmetry range greater than 1.5 cm indicating impaired ankle dorsiflexion.^34^ Both low and high ankle dorsiflexion range of motion^19^ and asymmetry ≥ 6.5º (approximately 1.8 cm)^16^ have previously identified recruits or trained military personnel at heightened risk of lower leg (knee and below) and/or musculoskeletal injuries. New Zealand Army recruits commencing training with high and low or asymmetry in ankle dorsiflexion range of motion may therefore be at greater risk of musculoskeletal lower limb injury (2.5 to 8 times, respectively^19^) however more research is required to directly confirm this relationship. While previous data has been based on male populations, the current study is one of the first to provide weight-bearing dorsiflexion lunge test values (ranges) for distance (cm) for both male and female army recruits upon entry to training.

Dynamic lower limb stability testing to identify baseline values and musculoskeletal injury risk is gaining popularity across sports and military populations. To our knowledge, this is one of few studies to investigate dynamic lower limb stability in male and female recruits. Our study found that although results from the Y Balance Test™ (Lower Quadrant) for anterior and posteromedial direction asymmetry were within normal limits (<4 cm and ≤ 4 cm respectively), New Zealand Army recruits exhibited high posterolateral reach asymmetry and low normalized composite reach scores; particularly female recruits. High posterolateral Y Balance Test™ asymmetry (≥4.08 cm) has previously been associated with approximately 5.5 times the risk of developing patellofemoral pain over 6 weeks of basic training.^21^ Approximately 51% of recruits in the current study, including 70% of the female recruits, displayed asymmetry greater than or equal to 4.08 cm.

The association between low baseline composite (normalized) reach score and injury risk in recruits has had limited previous investigation. No association was found between composite reach (measured using the Y Balance Test™) and injury prediction in U.S. military recruits^22^ although injury was reported as an incidence of pain and actual injuries were not reported. However, low normalized composite reach score (<94%; measured using the Star Excursion Balance Test) has been associated with 6.5 times greater risk of lower limb musculoskeletal injury in female basketballers.^20^ Approximately 60% of the recruits in the current study scored below this injury risk cut point, although interestingly, higher scores were reported for male and female New Zealand Army Officer trainees (96% male, 98% female) at entry to training.^40^ Due to the high prevalence of knee injury in New Zealand Army recruits,^2^ further research is required to identify if recruits, particularly females, may be at high risk of developing lower limb injury, such as patellofemoral pain, during the early weeks of basic training.

Slow 2.4-km run time, altered ankle dorsiflexion range of motion, and low lower limb dynamic stability have the potential to be modifiable injury risk factors. If deficits are identified prior to training, mitigation measures could be taken where possible to reduce likelihood of recruit musculoskeletal injuries during basic training.

### Strengths

To our knowledge, this is the first study to describe gender-specific profiles for personal, lifestyle, and physical performance characteristics of New Zealand Army recruits entering basic training and the sample size was robust. A combination of a self-reported questionnaire, simple field-friendly, cost and resource effective objective measures were used, which are ideal for mass screening and are repeatable throughout a military career. This study is one of few to describe results of the weight-bearing dorsiflexion lunge test using a simple validated distance method and potentially one of the first to report results for dynamic lower limb stability (balance) for male and female New Zealand Army recruits using the Y Balance Test™. Recruits participating with minor injuries were also included providing a real-world sample for physical performance testing. Our findings are generalizable to recruit populations from other militaries who display similar personal characteristics. Additionally, interoperability is enhanced by the sharing of military health information across different military populations and countries. Results of this study provide important baseline values which can be used for future studies of injury risk in army recruits.

### Limitations

Ethnic diversity information was not captured; therefore, the personal, lifestyle, and physical performance characteristics of different Maori, Pacific Island, European, and other populations applying to New Zealand Army basic training is not available. Female recruits were included, however numbers were small. Cut-off points for the Y Balance Test™ were based predominantly on research of musculoskeletal injury risk in sports populations as limited or no data is available for cut-off points for army recruits.

## CONCLUSION

Describing the profile of New Zealand Army male and female recruits at entry to basic training has provided baseline personal, lifestyle, and physical performance characteristic data. New Zealand Army recruits are predominantly young active males, few had preexisting injury in the previous year, pre-entry alcohol consumption was higher than recommended, and a minority are current smokers. The majority of recruits had low aerobic fitness, average ankle dorsiflexion range, and low dynamic lower limb stability. A number of these baseline values are associated with higher musculoskeletal injury risk and are potentially modifiable. Identified risk factors could be mitigated leading to lower recruit musculoskeletal injury and associated burdens during basic training. Describing the profile of recruits entering training is the first step in injury prevention and future research should investigate the association of baseline personal, lifestyle and physical performance measures to actual injuries sustained by recruits during training.
